# Lung cancer metastasis to oral soft tissues; Systematic review of 122 cases

**DOI:** 10.4317/jced.59773

**Published:** 2022-10-01

**Authors:** Sonia Gupta, Manveen-Kaur Jawanda, Neal-Bharat Kedia, Aayush-Ranjan Deb, Aruna Ganganna, Kumar Saurabh, Sumit-Kumar Yadav, Achla-Bharti Yadav

**Affiliations:** 1 MDS, Reader, Dept. of Oral Pathology and Microbiology & Forensic odontology, Rayat and Bahra Dental college and hospital, Mohali, Punjab, India; 2 MDS, Professor & Head, Dept. of Oral Pathology and Microbiology & Forensic odontology. Laxmi bai institute of dental sciences and hospital, Patiala, Punjab, India; 3 MDS, Professor & PG Guide, Dept. of Orthodontics & Dentofacial Orthopedics, Buddha Institute of Dental Sciences & Hospital, Patna, Bihar, India; 4 BDS, Intern, Sri Ramachandra Faculty of Dental Sciences, Porur, Chennai, Tamilnadu, India; 5 MDS, Assistant Professor, Department of Periodontology, JSS Dental College and Hospital, Mysore, Karnataka, India; 6 MDS, Medical Officer (Dental Surgeon), PHC, Bind, Bihar, IndiaMDS, Medical Officer (Dental Surgeon), PHC, Bind, Bihar, India; 7 MDS, Professor, Department of Orthodontics & Dentofacial Orthopedics, D J College of Dental Sciences & Research, Modinagar, UP, India; 8 MDS, Professor, Department of Oral Pathology & Microbiology, D J College of Dental Sciences and Research, Modinagar, UP, India

## Abstract

**Background:**

Lung cancer metastasis to oral region is very rare. Studies have been published analysing the cases of metastatic tumours to the oral cavity by many researchers. But very few research work has been conducted till date to analyse only the oral soft tissue metastasis from Lung cancer as the primary source. The goal of this study was to examine published cases of oral soft tissue metastasis from lung cancer as the only primary source from 1st August 1977 to 31st December 2021.

**Material and Methods:**

An electronic search of the published English literature was performed in PubMed/ Medline, Scopus, Google Scholar, and Research gate databases, using keywords like ‘Lung cancer’, OR/ AND ‘Lung carcinoma’ OR/ AND ‘Oral cavity’, OR/AND ‘Metastasis’, OR/AND ‘Primary’, OR/AND ‘Source’, OR/AND ‘Initial’, OR/AND ‘Tongue’, OR/AND ‘Palate’, OR/ AND ‘Tonsil’, OR/AND ‘Lip’, OR/AND ‘Buccal mucosa’, OR/AND ‘Floor of mouth’, OR/AND ‘Salivary glands’, OR/ AND ‘Parotid’, OR/ AND ‘Submandibular’, OR/ AND ‘Sublingual’ OR/ AND ‘Mandible’, OR/AND ‘Maxilla. We also searched all related journals manually. Reference list of all articles was also checked.

**Results:**

Our research revealed total 122 patients. The most prevalent diagnosed metastatic lung cancer was adenocarcinoma. Gingiva, tongue and tonsils were the most common site of metastasis. 54% patients died of metastasis with a survival time of 1 week to 2.5 years.

**Conclusions:**

Oral soft tissue metastasis from lung cancer has a bad prognosis. More cases need to be published in order to raise awareness of these lesions and gain a better understanding of their characteristics.

** Key words:**Lung cancer, metastasis, oral, primary, soft tissues.

## Introduction

GLOBOCAN databases has documented lung cancer (LC) as the 2nd most common diagnosed cancer leading to high mortality after breast cancer worldwide (1). The unique feature of LC is its long term asymptomatic clinical presentation and when the disease reaches in its advanced stages, it is associated with a high risk of developing distant metastasis. It has been found that most of the time, the symptoms are disregarded by the patients even once they arise, resulting in a delay in diagnosis and treatment (2). The most common regions of distant metastasis via LC in the body are liver, kidney, adrenals, brain, bones, scalp, and other organs (3). Oral cavity is the uncommon site of LC metastasis, and mostly affects soft tissues rather than jaw bones (JB) (4). The prognosis for such cases is poor, indicating the critical importance of their early identification and management. Due to their strong resemblance to some benign growths, late appearance, or lack of interpretation, diagnosis of these metastatic lesions remains difficult for clinicians and pathologists (5). Studies have been published analysing the cases of metastatic tumours to the oral cavity by many researchers. But very few research work has been conducted till date to analyse the lung cancer metastasis as the soul primary source particularly to the oral soft tissues (OST). The goal of this research was to examine published cases of oral soft tissue metastasis (OSTM) from LC as the sole primary source in the literature from 1st August 1977 to 31st December 2021, and to learn about their characteristics.

## Material and Methods

The Preferred Reporting Items for Systematic Reviews and Meta-Analyses (PRISMA) standards were used to conduct this research. There was no need to seek any ethical approval because of the nature of the current review.

- Focused PECO question

For conducting this review, we framed a focused PECO question ‘How many cases of OSTM from LC as the sole primary source have been documented in the literature and what is the prognosis of these metastatic lesions’?

Population: Patients with OSTM from LC

Exposure: Distant metastasis of LC

Comparator: Not applicable for current research

Outcome: Prognosis of OSTM from LC

**Figure 1:**
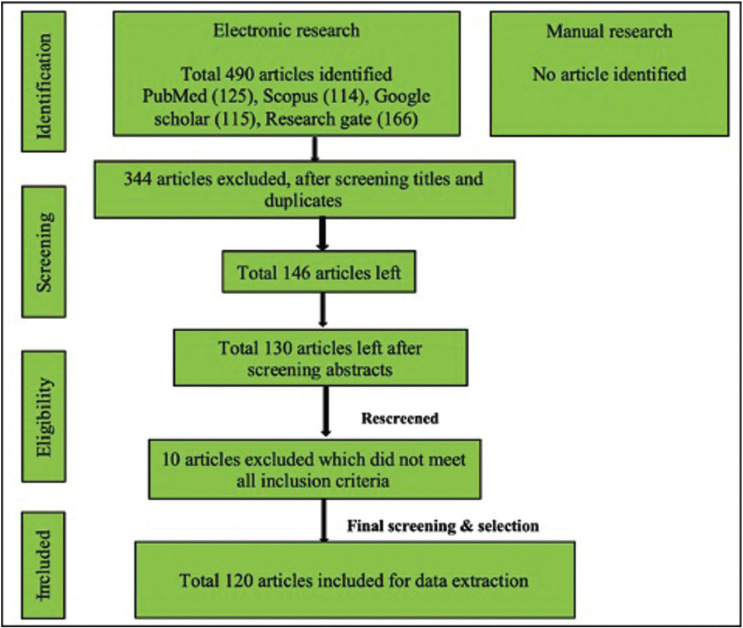
PRISMA flowchart showing search strategy.

- Search strategy for identification of studies (Fig. 1)

An electronic search of the published English literature was performed in PubMed/ Medline, Scopus, Google Scholar, and Research gate databases, using keywords like ‘Lung cancer’, OR/ AND ‘Lung carcinoma’ OR/ AND ‘Oral cavity’, OR/AND ‘Metastasis’, OR/AND ‘Primary’, OR/AND ‘Source’, OR/AND ‘Initial’, OR/AND ‘Tongue’, OR/AND ‘Palate’, OR/ AND ‘Tonsil’, OR/AND ‘Lip’, OR/AND ‘Buccal mucosa’, OR/AND ‘Floor of mouth’, OR/AND ‘Salivary glands’, OR/ AND, Mandible’, OR/AND ‘Maxilla’. We also searched all related journals manually. Reference list of all articles was also checked.

- Screening of studies

The current review involved three steps screening of the studies. In the first step, titles were reviewed by two authors (SG, MKJ) independently and duplicates were removed. Then four authors ( NBK, ARD, AG, KS) reviewed the selected abstracts of all the reports independently. In the final stage, the text of selected studies was screened by authors separately (SKY, ABY). Full report was collected, discussed, and resolved for cases among all authors that appeared to fit the inclusion criteria or for which evidence was insufficient to make a clear determination.

- Inclusion criteria

• Confirmed cases of OSTM from LC as the sole primary source. Papers included were from 1st August 1977 to 31st December 2021.

• Type of studies: Case reports, letter to editor, Short communication, Retrospective analysis and correspondence.

• Cases were selected beyond the restriction of limitations on parameters such as age, gender, ethnicity or socioeconomic status, etc.

• Articles published only in English language were included.

- Exclusion criteria

• Cases with no definite diagnosis of OSTM from LC as the sole primary source.

• Publications reporting the OSTM from any other site than lung.

• Cases of Jaw bone metastasis from LC as the primary source.

• Other epidemiological studies, cross-sectional studies and cohort studies were excluded as they did not provide individual patient’s data.

• Review articles, editorials, conference abstracts, hypothesis papers, web news, media reports, animal studies.

- Outcome measures

1. Primary outcome measures: To evaluate the number of cases of OSTM from LC as the sole primary source reported in the literature and to determine their prognosis.

2. Secondary outcome measures: To evaluate factors such as: World-wide distribution of cases of OSTM from LC, Patient’s demographic details, Associated risk factors, Predominant site of OSTM from LC, Clinical features of these metastatic lesions, Most prevalent type of metastatic LC and Type of therapies used to treat such metastatic lesions.

- Risk of bias assessment 

Most of the studies included in this review were case reports. Risk of bias in the included studies were appraised following CARE checklist guidelines. In many of the studies, there was missing information regarding many parameters used for data extraction in our research. We tried reaching the authors of those cases to clarify this bias; however we were unable to recover the missing information.

- Data extraction & analysis

After study selection, screening and a thorough examination, the data were extracted. The information gathered was cross-checked and tabulated into three Tables (Table 1- Table 3 cont.-2). In case of missing data, 6 weeks’ time was given to gather the information. If the information was still missing, we then indicated the missing data as “Not available (NA)” in the text and in the Tables.

**Table 1:**
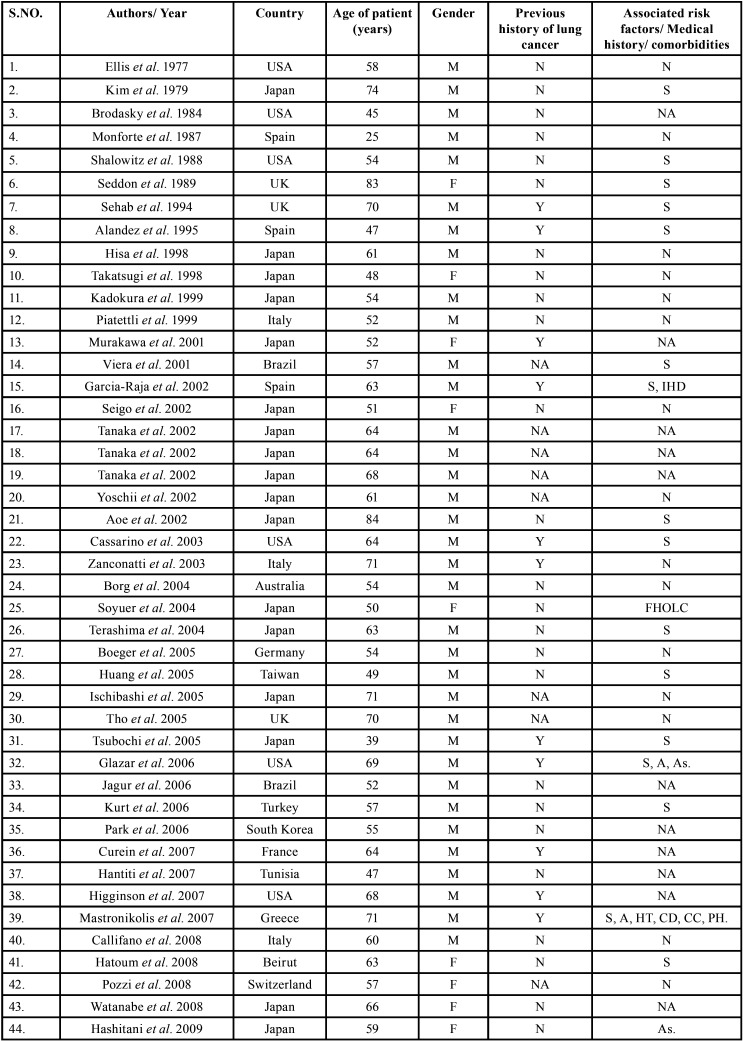
Demographic data of patients with oral soft tissue metastasis from lung cancer as the sole primary source (1st August 1977 to 31st December 2021).

**Table 1 cont.**
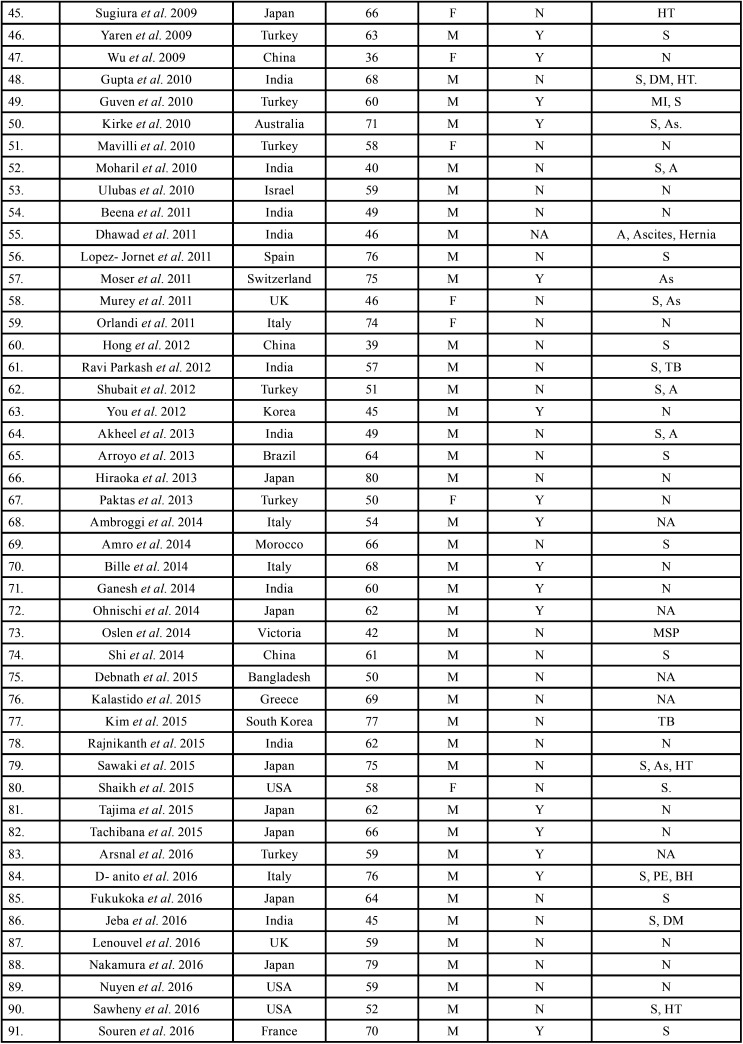
Demographic data of patients with oral soft tissue metastasis from lung cancer as the sole primary source (1st August 1977 to 31st December 2021).

**Table 1 cont.-1:**
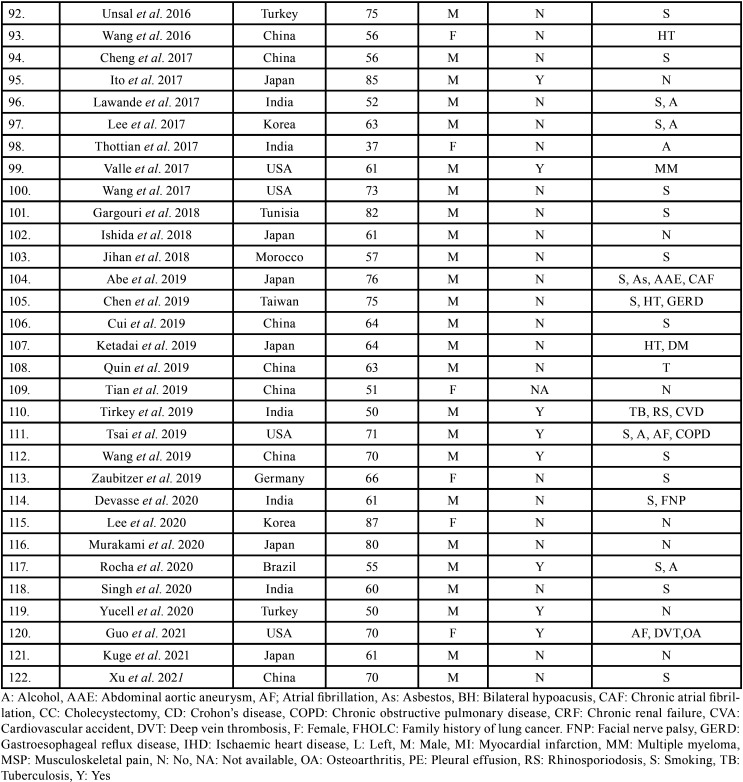
Demographic data of patients with oral soft tissue metastasis from lung cancer as the sole primary source (1st August 1977 to 31st December 2021).

**Table 2:**
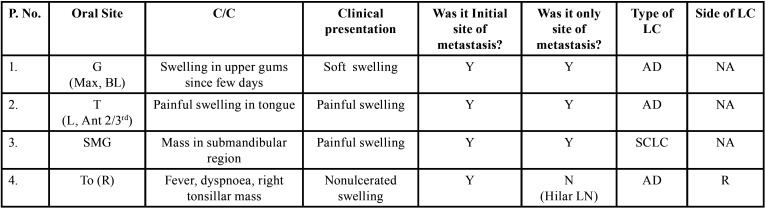
Clinical details of patients with oral soft tissue metastasis from lung cancer (1st Aug 1977 to 31st December 2021).

**Table 2 cont.**
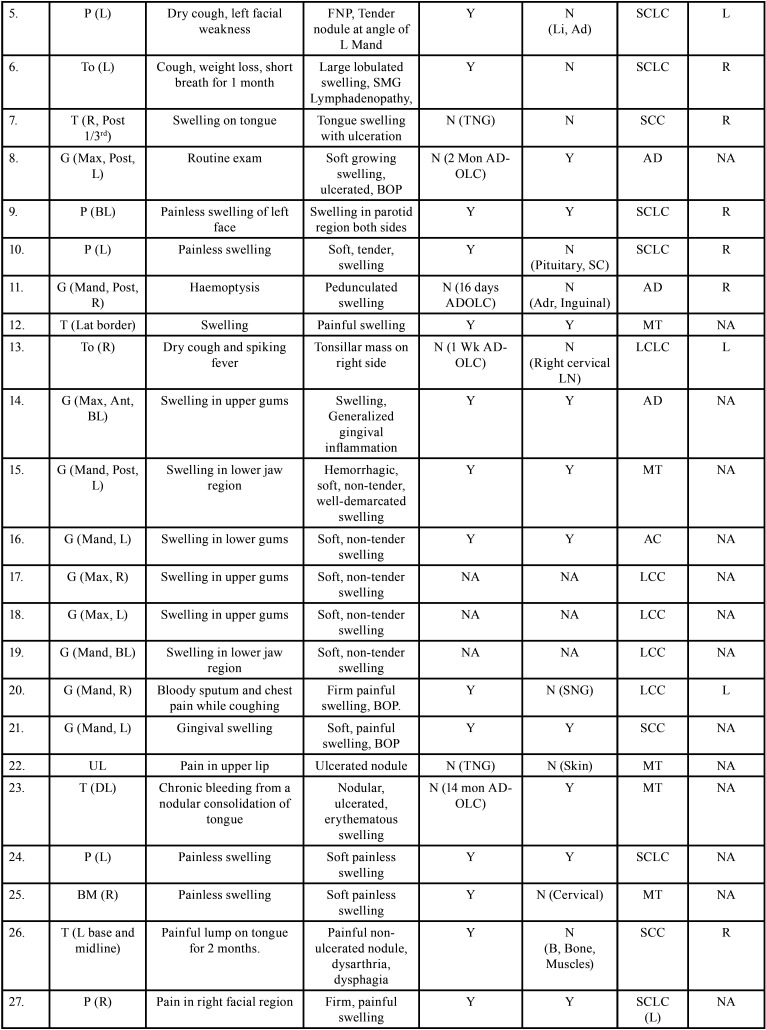
Clinical details of patients with oral soft tissue metastasis from lung cancer (1st Aug 1977 to 31st December 2021).

**Table 2 cont.-1:**
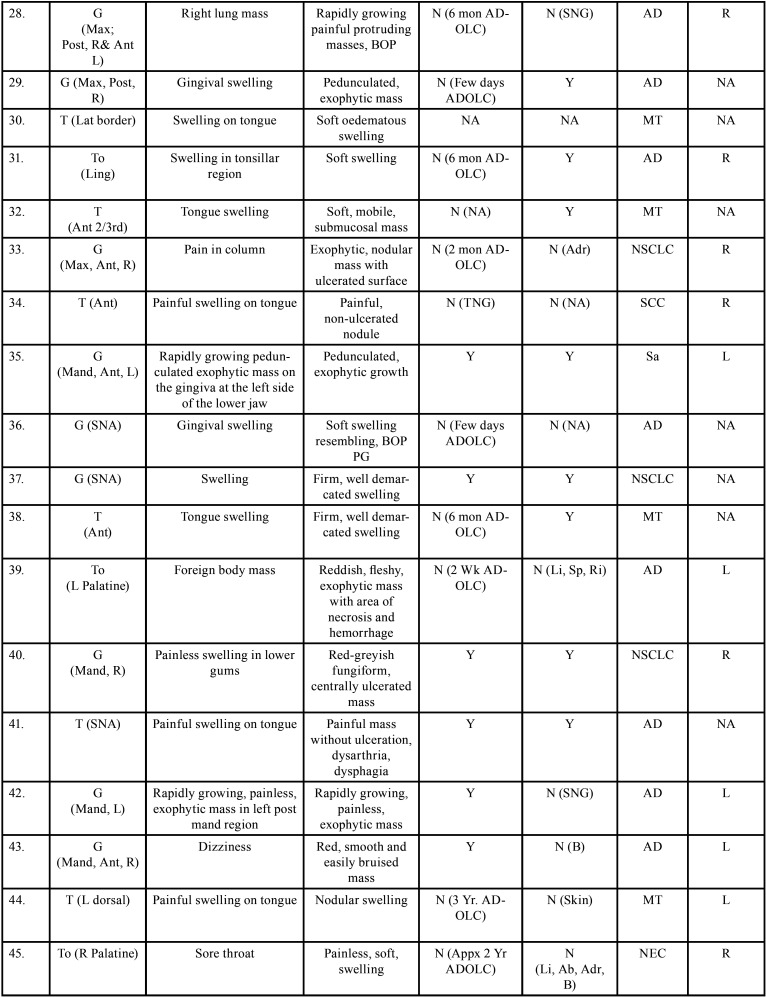
Clinical details of patients with oral soft tissue metastasis from lung cancer (1st Aug 1977 to 31st December 2021).

**Table 2 cont.-2:**
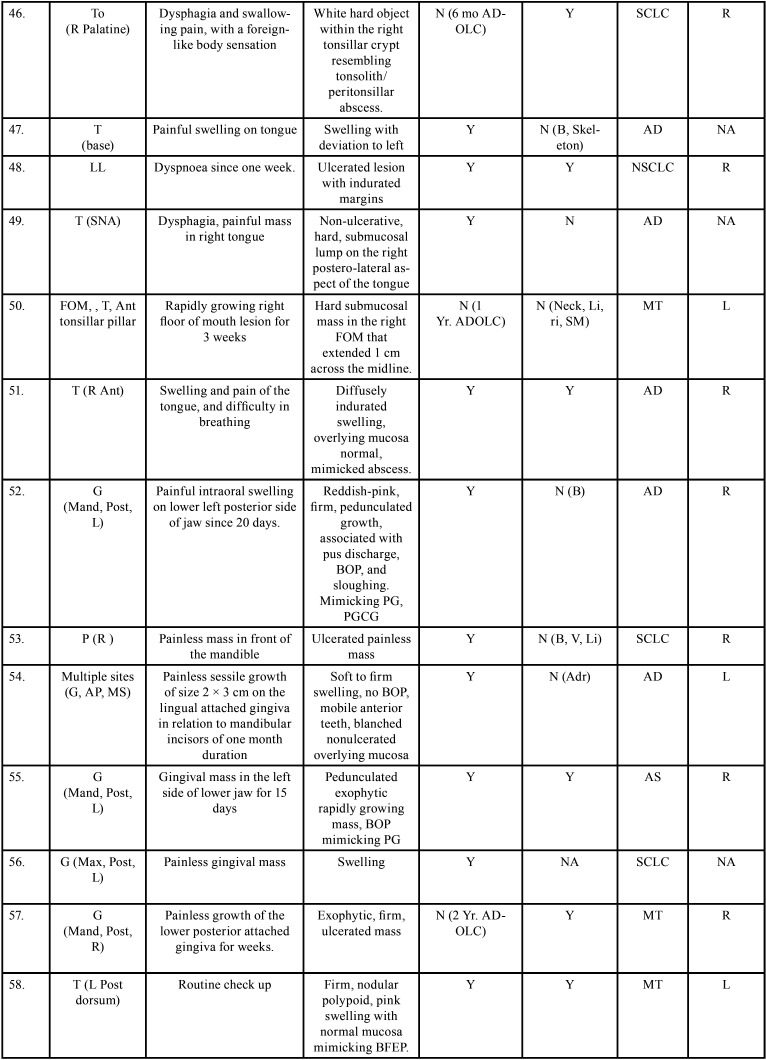
Clinical details of patients with oral soft tissue metastasis from lung cancer (1st Aug 1977 to 31st December 2021).

**Table 2 cont.-3:**
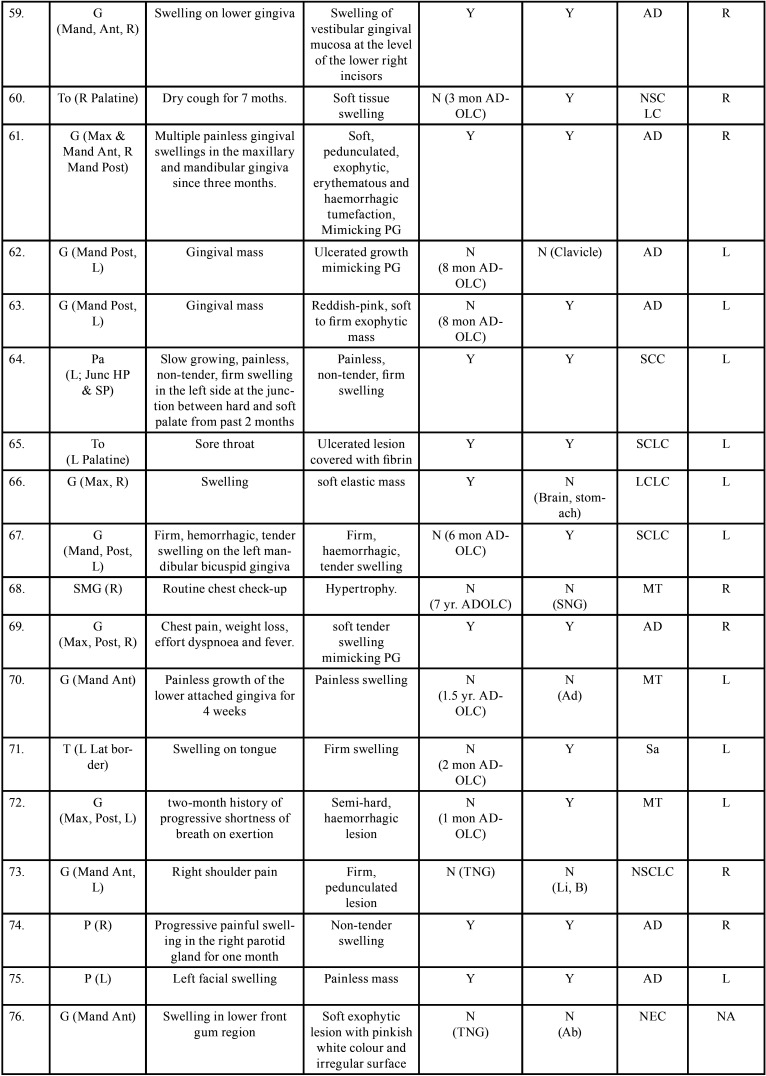
Clinical details of patients with oral soft tissue metastasis from lung cancer (1st Aug 1977 to 31st December 2021).

**Table 2 cont.-4:**
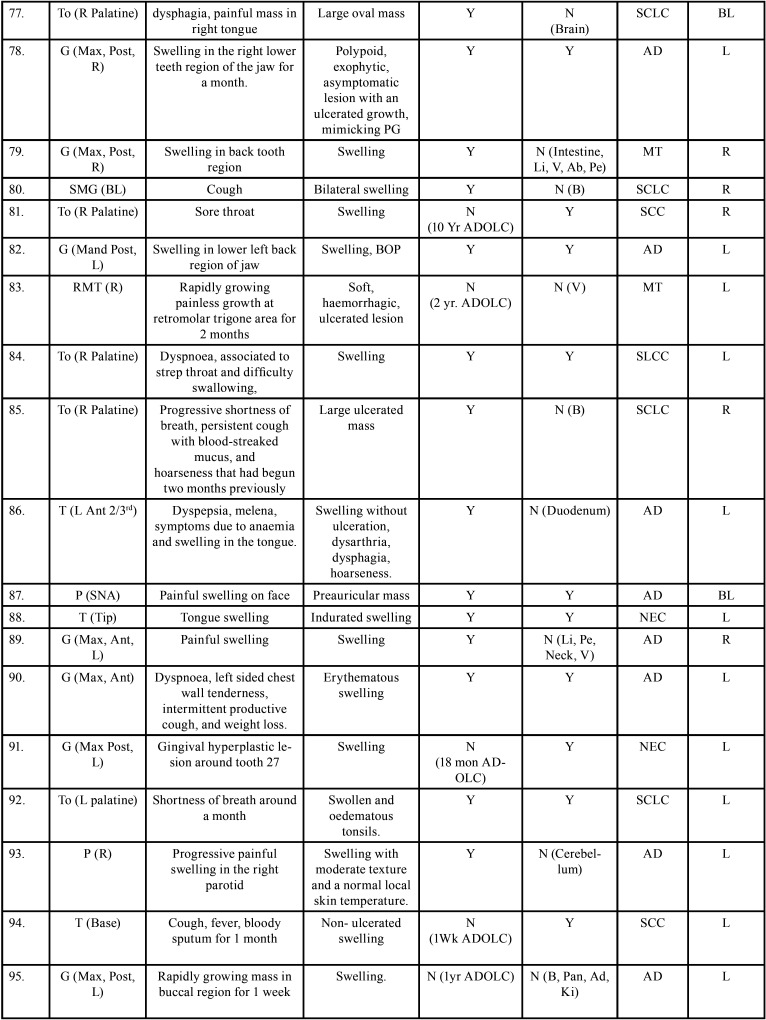
Clinical details of patients with oral soft tissue metastasis from lung cancer (1st Aug 1977 to 31st December 2021).

**Table 2 cont.-5:**
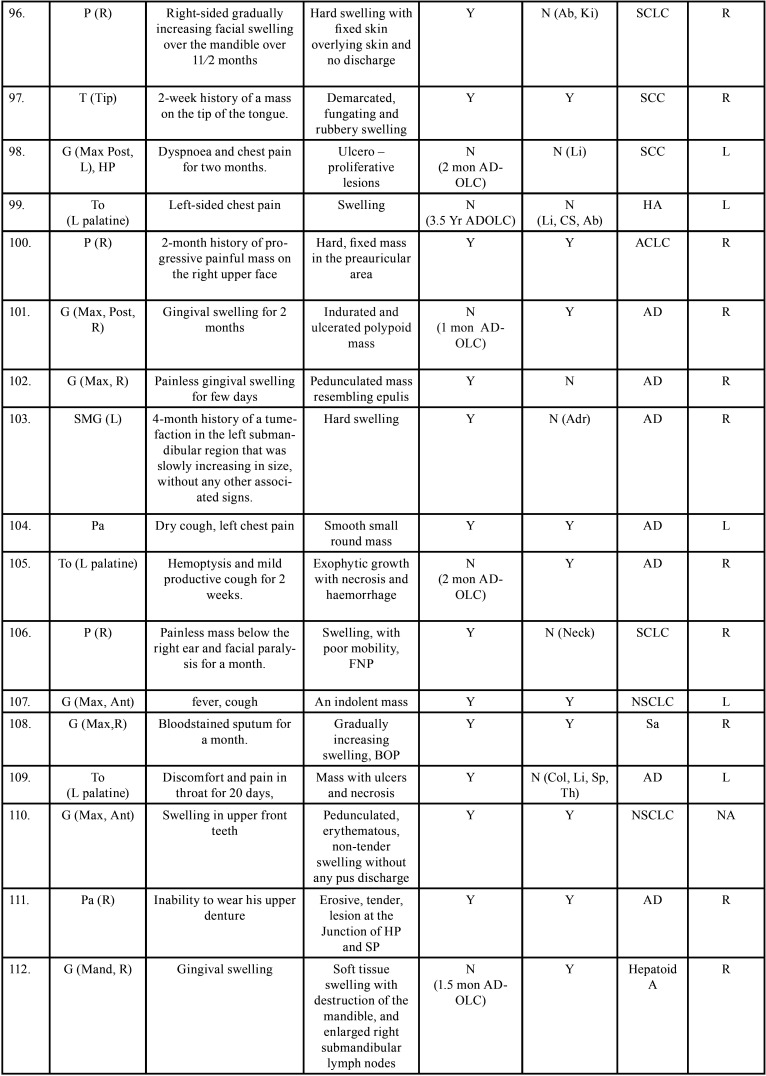
Clinical details of patients with oral soft tissue metastasis from lung cancer (1st Aug 1977 to 31st December 2021).

**Table 2 cont.-6:**
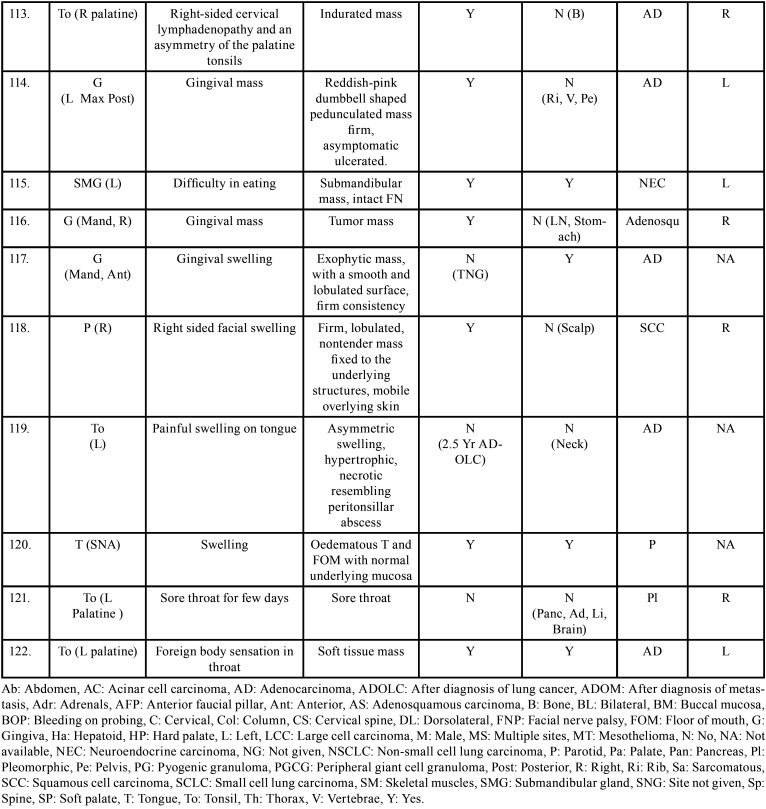
Clinical details of patients with oral soft tissue metastasis from lung cancer (1st Aug 1977 to 31st December 2021).

**Table 3:**
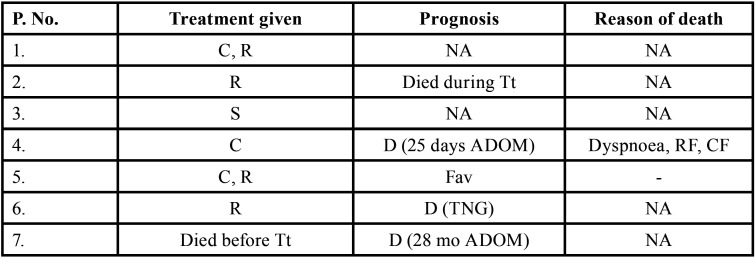
Data describing treatment and prognosis of patients with oral soft tissue metastasis from lung cancer (1st August 1977 to 31st December 2021).

**Table 3 cont.**
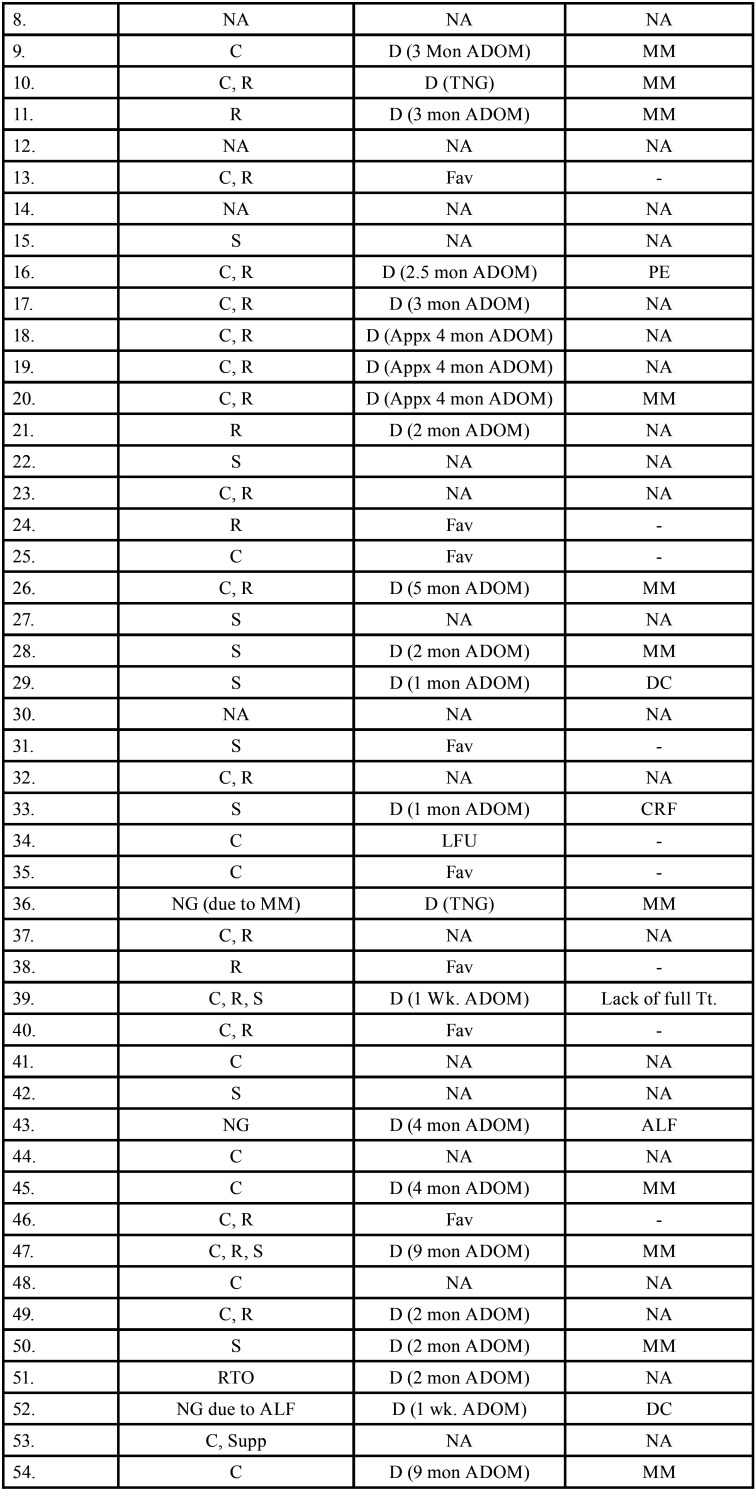
Data describing treatment and prognosis of patients with oral soft tissue metastasis from lung cancer (1st August 1977 to 31st December 2021).

**Table 3 cont.-1:**
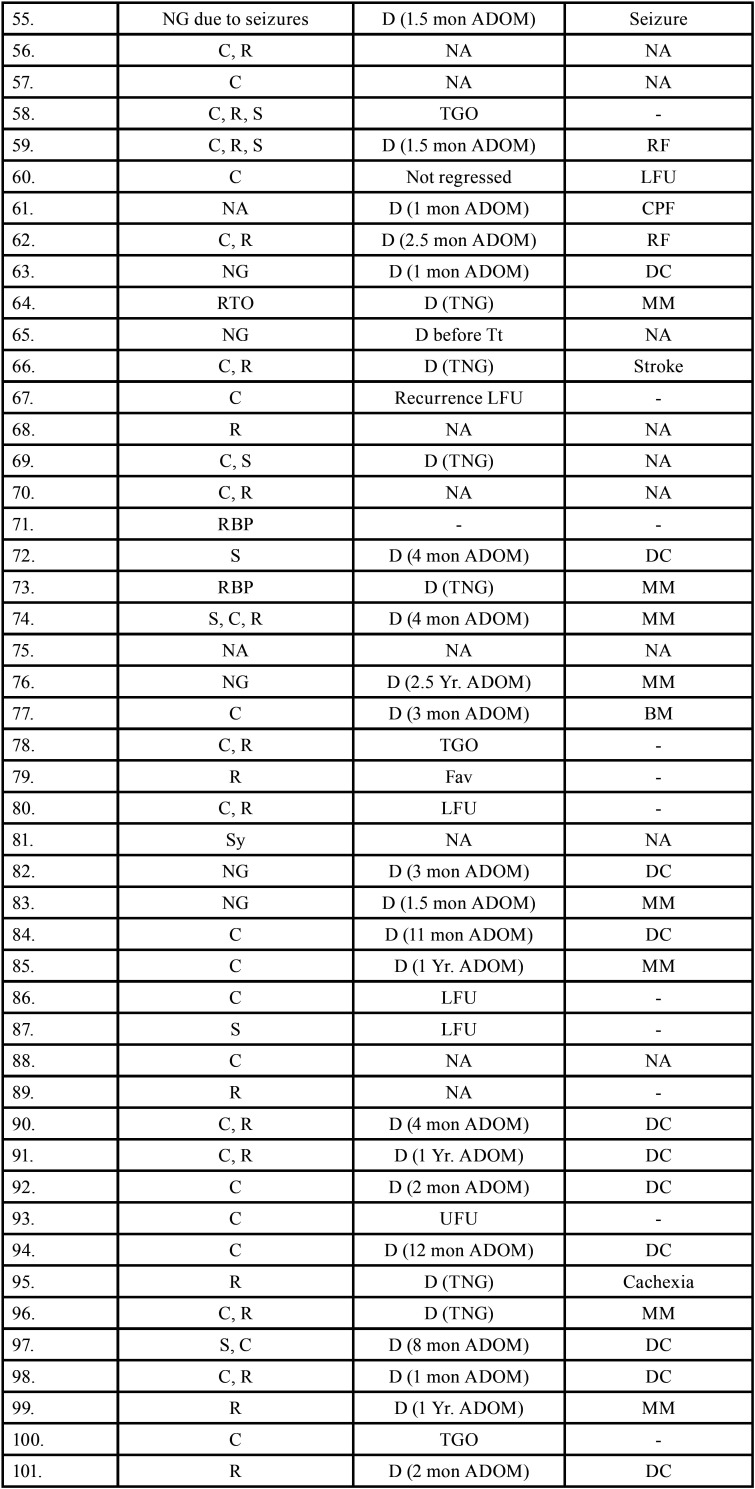
Data describing treatment and prognosis of patients with oral soft tissue metastasis from lung cancer (1st August 1977 to 31st December 2021).

**Table 3 cont.-2:**
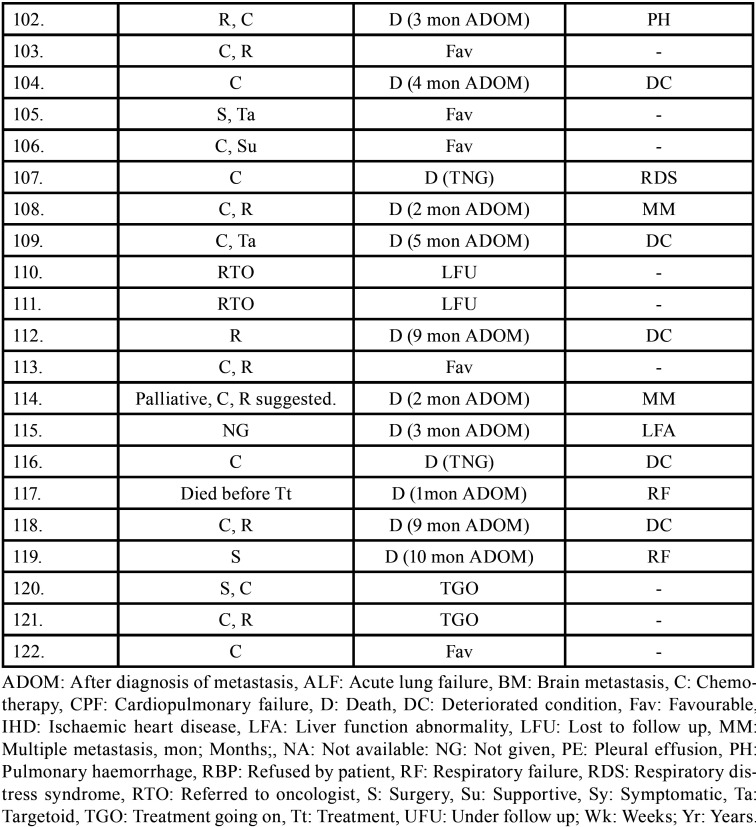
Data describing treatment and prognosis of patients with oral soft tissue metastasis from lung cancer (1st August 1977 to 31st December 2021).

Results (Table  Table 4,  Table 4 cont.-1)

**Table 4:**
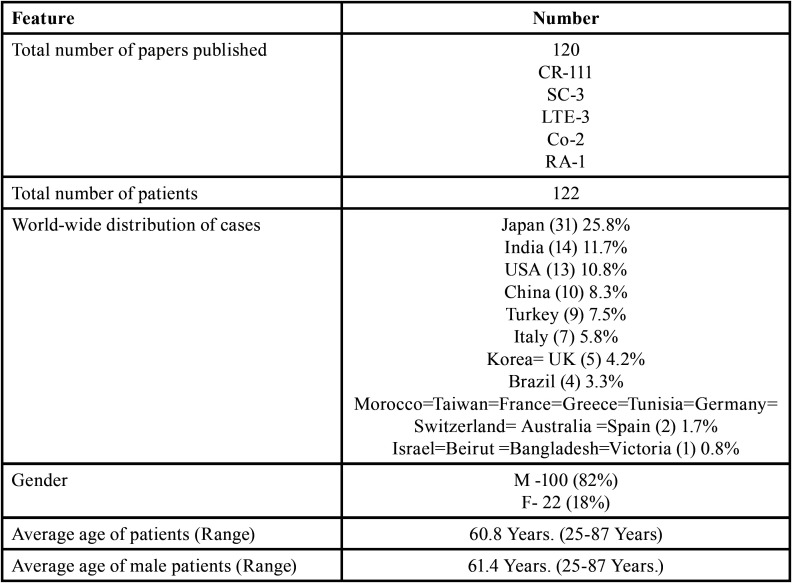
Summary of results documented from literature research describing the characteristics of oral soft tissue metastasis from lung cancer (1st August 1977-31st Decemebr 2021).

**Table 4 cont.**
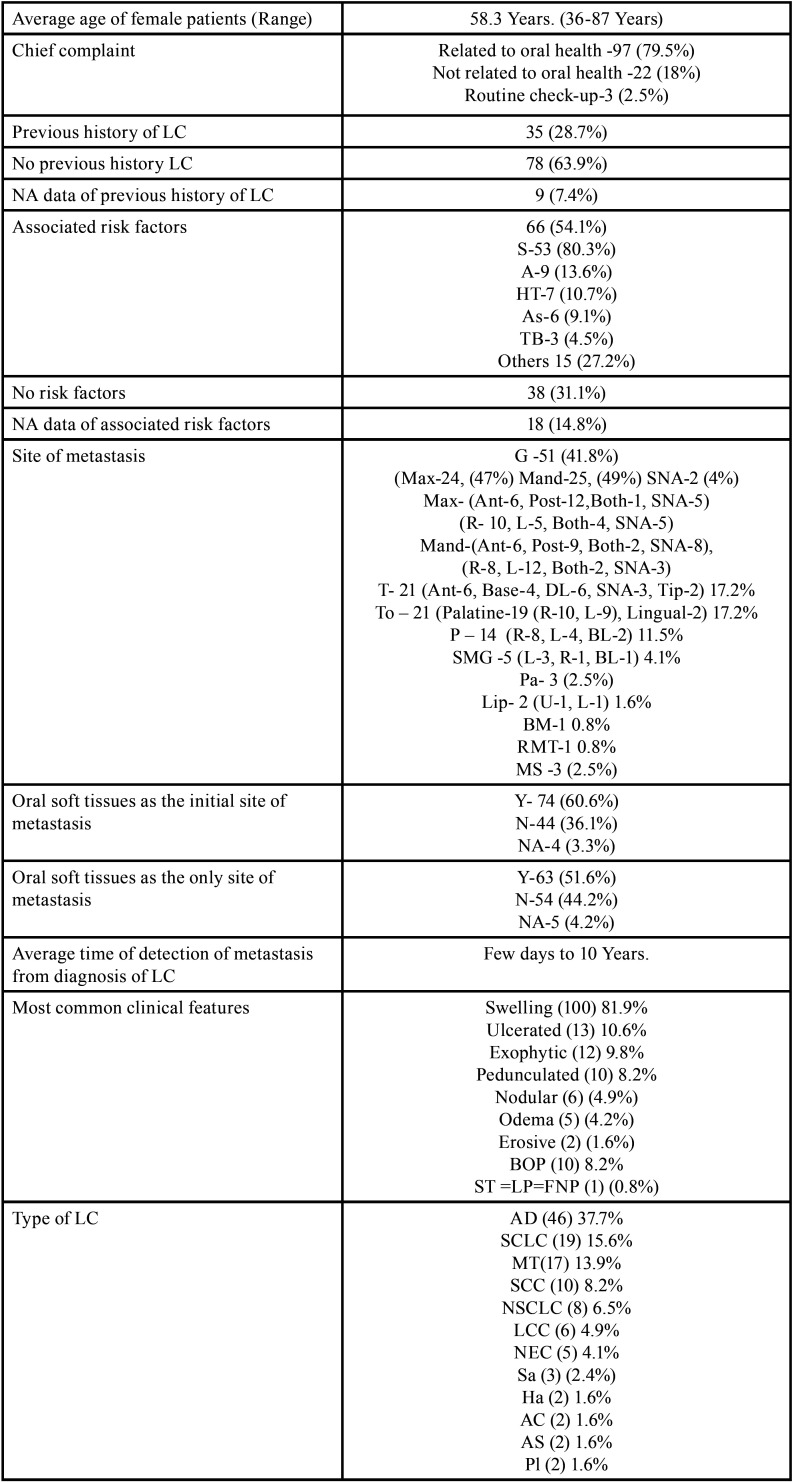
Summary of results documented from literature research describing the characteristics of oral soft tissue metastasis from lung cancer (1st August 1977-31st Decemebr 2021).

**Table 4 cont.-1:**
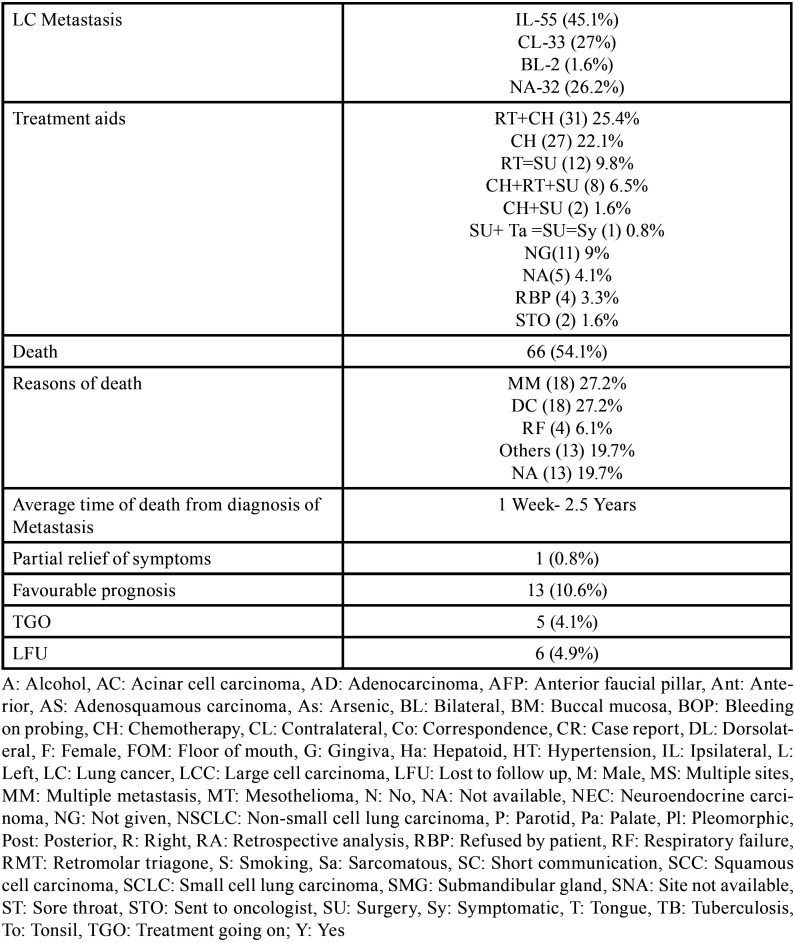
Summary of results documented from literature research describing the characteristics of oral soft tissue metastasis from lung cancer (1st August 1977-31st Decemebr 2021).

Results were expressed in descriptive statistics. There were 111 Case reports, 3 Letter to editor, 3 Short communications, 2 Correspondences, and 1 Retrospective analysis. There were 122 patients in total, with 100 males (82%) and 22 females (18%). The maximum number of cases were from Japan (n-31), India (n-14), and USA (n-13). The patients’ average age was 60.8 years (range 25-87). Mean age was 61.4 years in males and 58.3 years in Females. 35 of the 122 patients (28.7%) had a previous history of LC, while the other 78 had none (63.9%) . 54.1% had associated risk factors or underlying comorbidities. Most OSTM metastasis was found in Gingiva (41.8%)>tongue = tonsils (17.2%)>parotid glands (11.5%)>submandibular glands (4.1%))> palate (2.5%)>lip (1.6%)>retromolar trigone = buccal mucosa (0.8%). Patients presented with variable clinical features. OST were the initial site of metastasis in 60.6% individuals. OST were the only site of metastasis in 51.6% of cases, whereas 44.2% cases involved other parts of the body also. The most common type of LC diagnosed was Adenocarcinoma (n-46). The most common treatment aids included combined Radiotherapy and chemotherapy (25.4%), followed by chemotherapy alone (22.1%). 66 individuals (54.1%) died with a mean survival rate of 1 week to 2.5 years.

## Discussion

Metastasis to the oral cavity is a rare occurrence, with the real incidence unclear (1-2% of all oral cancers). Because of their rarity, they are sometimes overlooked for a long time before being discovered and are diagnosed during investigations. According to epidemiological investigations, LC is the most common primary source of OSTM, while Breast cancer is the most common source of JBM (5). In this study, we found 122 documented cases of OSTM from LC as the sole primary source.

Studies reveal that OSTM affects both genders equally with peak incidence of 5th- 6th decade with a male majority (5). In the current study also, there was a male predominance, with M: F = 4.5:1 with age from 2nd-8th decade. According to studies, Smoking and tobacco consumption habits are strongly linked to the development of LC. Nicotine and its derivatives, which are found in tobacco and smoke, help to promote the expression of oncogenic proteins which leads to the spread of cancer (6). In our study, 80% cases had habit of smoking. Asbestos-induced inflammation has been found to promote the neoplastic transformation of mesothelial cells leading to development of LC, particularly mesothelioma (7,8). In the present research appx 9% patients had a history of asbestos exposure.

People with underlying comorbidities are more likely to acquire cancer and have a worse prognosis as a result of distant metastasis induced by a weakened immune system. 27.2% patients in this study had a variety of underlying comorbidities, the most prevalent of which were cardiac, respiratory, and renal.

The incidence of oral metastatic lesions is not so specific in any region of the world. In our study, the maximum number of cases were from Japan (n-31), followed by India (n-14), and USA (n-13), Various other regions were also involved (Table 4). Looking at this data, wide region involvement of OSTM from LC can be appreciated.

The most common site of OSTM is the attached gingiva (57%), followed by the tongue (27%), tonsil (8%), palate (4%), lip (3%), Buccal mucosa (1%), and floor of mouth (<1%) (9). In the present research also, some similar results were observed with maximum cases found on gingiva >tongue = tonsils.

Pathogenic mechanisms of OSTM aren’t completely understood. Metastasis is a multistage process that involves tumour cells being detached from their originating site and being transported to a secondary site via lymphatic or hematogenous channels (10). One of the proposed pathways is the “Batson’s plexus,” a valveless prevertebral venous plexus network that involves retrograde tumour cell movement from the lungs to the face. Another pathway of metastasis in LC involves direct suction, access to the pulmonary vein, and drainage to the left side of the heart (11).

Chronically, inflamed mucosa in the oral soft tissues, particularly attached gingiva, contains a dense capillary network that can trap malignant cells and promote metastases. On the other hand, some reports of metastases to the post-extraction site suggest that local variables in the extraction or wound area may attract circulating tumour cells. Because tooth extraction generates a milieu rich in growth factors, it may promote metastasis. The extraction site of a tooth is thought to be a microenvironment rich in local growth factors that encourage metastatic cell development (9).

Studies conclude that gingival metastasis mostly occurs in mandibular area than maxillary with predominancy of posterior side involvement (5,9,10). In the current research however, there was almost equal involvement of maxillary and mandibular gingiva. Posterior region was predominantly affected than anterior region. OSTM usually occurs unilaterally. In our research, left side predominated as compared to right in the mandibular gingiva, while maxillary gingiva showed right side predominance. 2 cases showed BL involvement each in mandible and 4 in maxilla.

The tongue is also a highly circulatory organ, which creates ideal conditions for the spread of cancer. According to a study, the lung was the second most common primary site metastasized to the tongue (5). The literature rarely mentions LC metastases to the tongue (12-14). In the present research, we could find 21 cases of tongue metastasis from LC. Posterolateral and dorsal part are more often involved in metastasis due to rich capillary and lymphatic network and immobility. On contrary, in the present research most of tongue metastasis was observed in anterior and dorsolateral region and base.

Tonsil metastasis has been reported to be extremely infrequent among oral soft tissues. Only 0.8 percent of 1535 cases of malignant palatine tonsillar tumours were metastases from an extra-tonsillar source, according to a study (15). In the current literature, 21 cases of tonsillar metastasis from LC have been observed and 19 of them were palatine tonsils. Only 2 cases occurred in lingual tonsil. Lymphatic spread to tonsils is rare due to lack of afferent lymphatic capillaries except retrograde spread via cervical lymph nodes or direct spread.

Metastatic deposits in the parotid gland from tumours outside the head and neck region are uncommon, but they have been recorded from lung, breast, and kidney cancers. These primaries account for roughly 10%–20% of all parotid secondary tumours (16). The abundant lymphatic flow seen in and around the gland parenchyma is most likely to blame for the dissemination to lymph nodes. Literature has revealed very rare number of cases of LC metastasis to parotid (17-18). In the current research out of 122 cases, only 14 involved parotid gland. Metastasis to other salivary glands such as submandibular gland, and sublingual gland is very rare. Owing to the lack of lymph nodes in these glands, route of metastasis is predominantly completely haematogenous. In our research, only 5 cases of submandibular gland metastasis from LC have been documented, while no case involved the sublingual gland.

The most common malignant neoplasms of the palatal mucosa are known to be minor salivary gland tumors (19). and metastatic tumors from a distant organ in this region is uncommon. In the present research, out of 122 cases of LC metastasis, only 3 were found in the palate region. Lip metastasis from distant resources is rarely documented in the literature. Few cases have been reported from colon and gastric cancers (20-21). In the present review, only 2 cases of lip metastasis from LC were notified. Floor of mouth and buccal mucosa are other rare sites of cancer deposits. Thin and movable mucous membrane in the floor of mouth region allows easy entrapment of tumour cells. In the present research, only one case of buccal mucosa and floor of mouth metastasis from LC was diagnosed.

Clinically, OSTM tumours grow rapidly causing pain, difficulty in chewing, dysphagia, disfigurement, and bleeding on probing . These metastatic lesions are often difficult to diagnose because their variable features such as polypoid or exophytic, highly vascularized growths or swelling, bear close resemblance to some benign hyperplastic or reactive oral lesions. Biopsy becomes mandatory to identify the exact primary source of metastasis. In present research, swelling was the most predominant clinical feature observed (81.9%). Other lesions appeared as ulcerative, exophytic, pedunculated, nodular, edematous, and erosive.

A history of LC could help in the detection of secondary metastatic cancer. Before the metastatic spread to the oral cavity, the majority of patients are aware of their primary tumours. However, metastasis to oral soft tissue via LC is a late indication. Only 35 of the patients in this research were aware of previous LC, whereas the other 78 had no such history.

Oral metastatic tumours are of high clinical importance because, they may be the only symptom of an undiagnosed underlying malignancy or the first sign of the metastasis. In our study,74 patients had evidence of metastasis as the initial symptom of the disease, whereas in 44 cases, metastasis was detected after the diagnosis of LC with an average time of few days to 10 years.

Histopathological examination is required to provide a conclusive diagnosis of the type of metastatic lesion. However, it might be difficult to make an exact diagnosis because these lesions have a varied histological appearance rather than a distinct picture. When the major focus of the primary metastatic site is known, diagnosing the secondary metastasis can be simple. Other tools, such as special staining, immunohistochemistry, and electron microscopy, may be necessary in some circumstances to determine the initial tumor’s nature.

LC has been divided into subgroups based on histopathology. Many new entities have recently been introduced to the 2015 World Health Organization classification system (22). Adenocarcinoma has been discovered to be the most prevalent kind of LC that metastasizes to the oral soft tissues. And same was the finding in this study as well.

Although LC entails multiorgan distant metastases, oral soft tissues might occasionally be the only site of metastasis. Out of 122 instances in this study, 63 had oral soft tissues as the only location of LC metastasis, whereas 54 had metastasis to other parts of the body as well such as brain, kidney, adrenal, liver, vertebrae, spine, pelvis, skin, and skeletal muscles.

Treatment and prognosis of oral metastatic lesions are determined by the site of genesis and the extent of the disease. Treatment options include biopsy, surgery, chemotherapy, radiotherapy, brachytherapy, and/or combination therapy. The most commonly used therapy aids in this study were chemotherapy and radiotherapy. Combination therapy was also used in many cases. Unfortunately, OSTM by LC has a bad prognosis with a maximum survival rate of 5 years. In some cases, a patient’s terminal stage of disease results in a loss of follow-up or, death. Even after treatment, 66 people died, according to the current study. The duration between death and oral metastasis diagnosis ranged from 1 week to 2.5 years. Multiple metastasis, deteriorated systemic condition, pleural effusion, and acute lung failure were the most common causes of death. 15 patients had a good prognosis with no signs of recurrence.

Limitations of the current study

One of the limitations of current research was involvement of only individual based studies such as case reports and case series. We could find a very few large-scale studies related to the subject in our search. Studies such as epidemiological, case control, cohort, etc. were excluded, because we also aimed to evaluate individual features of these metastatic lesions. And in those studies, individual data of patients was not available.

## Conclusions

During the last 44 years (Aug 1977-Dec2021), we found only 122 cases of OSTM rom LC as the sole primary source. This signifies a rare occurrence of OSTM from LC. The prognosis was poor involving 66 deaths out of 122 cases with a survival rate of 1 week to 2.5 years. Gingiva, Tongue and tonsils were the most prevalent oral soft tissues to get metastasized. Because of their resemblance to other pathologies, and late clinical signs, these lesions go unnoticed the majority of the time. Diagnosis of oral metastatic lesions is a challenging task for the clinicians, and pathologists. A thorough examination of the metastatic lesions is required, including a review of the patient’s medical history, clinical presentation, and early diagnosis in order to identify the primary site of metastasis and choose the best course of treatment. More cases need to be published in order to raise awareness of these lesions and gain a better understanding of their characteristics.
